# The Influence of Hobby Engagement on Cognitive Function Among Older Adults: A Population-Based Cohort Study Using Statistical Analysis and Machine Learning Predictions

**DOI:** 10.3390/neurolint17120192

**Published:** 2025-11-27

**Authors:** Yaxin Han, Shuo Li, Li Zesheng, Renzhi Tian, Chengchang Pan, Honggang Qi

**Affiliations:** 1 School of Computer Science and Technology, University of Chinese Academy of Sciences, Beijing 101408, China; hanyaxin@bjmu.edu.cn (Y.H.);; 2 School of Public Health, Peking University, Haidian District, Beijing 100191, China; 3 Catherine McAuley School of Nursing and Midwifery, University of Cork, T12YN60 Cork, Ireland; 124102231@umail.ucc.ie

**Keywords:** hobby engagement, cognitive decline, longitudinal studies, trajectory analysis, machine learning, aged, cognitive reserve, English longitudinal study of ageing (ELSA)

## Abstract

Background: Cognitive decline is an escalating public health challenge with global population aging. Understanding the association between hobby engagement and cognitive function is crucial for developing effective interventions. Methods: Utilizing data from waves 2 to 9 of the English Longitudinal Study of Ageing (ELSA), this study included 6854 community-dwelling adults aged 50 years and older. To comprehensively capture this relationship, we employed a multi-method analytical approach, encompassing multiple linear regression, linear mixed-effects models, group-based trajectory modeling (GBTM), and a comparison of machine learning algorithms to assess both associations and predictive performance. Results: Cross-sectionally, hobby engagement was significantly associated with higher baseline global cognitive, memory, and executive function scores. Longitudinally, hobbies were associated with a slower rate of decline in orientation, but not in memory, executive function, or global cognition. Group-based trajectory modeling, which identifies distinct subgroups with heterogeneous cognitive pathways, revealed that hobby engagement was associated with a substantially lower risk of belonging to a “persistently low cognitive function” trajectory (OR = 0.46). Among predictive models, the Scikit-learn Gradient Boosting Regressor demonstrated superior performance (RMSE = 0.7517, R^2^ = 0.3033), outperforming deep learning approaches. Conclusions: Our findings suggest that hobby engagement may have domain-specific protective effects on cognitive health in older adults, most notably by drastically reducing the risk of a severely adverse cognitive trajectory. Promoting hobby participation therefore emerges as a potential viable, low-cost, and impactful public health strategy for preserving cognitive health in aging populations.

## 1. Introduction

With the aging of the population, cognitive decline is increasing as a public health problem. The World Health Organization (WHO, 2022) estimates that 55 million people are living with dementia today, with 10 million new cases arising each year, which means one every three seconds. This trend underscores the urgent need for early identification and effective intervention for cognitive decline [1]. Although cognitive decline involves complex neurobiological mechanisms [2], a large body of epidemiological and experimental evidence suggests that modifiable lifestyle factors [3], such as regular physical activity [4], social interaction [5] and ongoing cognitive stimulation [6], play a fundamental role in increasing cognitive reserve and delaying memory decline. In this context, systematically exploring the long-term association between hobbies and cognitive function in older adults, not only deepens our understanding of cognitive protective mechanisms but also informs the development of effective health promotion strategies. However, elucidating this complex relationship requires analytical approaches that go beyond conventional methods.

Previous research has been predominantly focused on pathological or risk factors, such as depression [7,8], sedentary behavior [9], falls [10,11], hearing loss [12,13]. In contrast, positive health behaviors like hobby engagement have received inadequate attention.

A survey from China confirmed a cross-sectional association between social activity participation and cognitive function [14]. However, without follow-up data, this study could not examine cognitive changes over time or capture their dynamic nature. The heavy reliance on cross-sectional studies, which capture a single time point, makes it difficult to track dynamic cognitive changes. Consequently, systematic evidence on longitudinal cognitive trajectories remains scarce. Similarly, a recent analysis of data from the Canadian Longitudinal Study on Aging (CLSA) found that certain physical activities and better self-rated health were associated with memory improvement, but the relatively short follow-up period precluded insights into the long-term patterns of cognitive decline. Furthermore, the generalizability of findings on cognitive decline is often limited due to underrepresented populations and inconsistent conclusions across studies [7].

Furthermore, the field has been hampered by methodological limitations. There remains a scarcity of dynamic modeling approaches capable of identifying heterogeneous subgroups with distinct cognitive trajectories. Similarly, the development of high-precision predictive models leveraging machine learning for early identification of at-risk individuals is notably lacking, which hinders timely intervention. Looking ahead, the emergence of neural interfaces and brain–computer interfaces (BCIs) offers a transformative avenue for capturing real-time neural signatures of cognitive states [15]. Integrating such neurotechnological perspectives could not only revolutionize daily cognitive monitoring but also deepen our understanding of the hobby-cognition link at a neurophysiological level.

To address these gaps, the present study leverages the long-term, population-based ELSA cohort and adopts an integrative, multi-method analytical framework. We aim to (1) examine cross-sectional and longitudinal associations of hobby engagement with the rate of cognitive decline; (2) identify heterogeneous subgroups following distinct cognitive trajectories and the influence of hobbies thereon; and (3) develop and compare machine learning models to predict cognitive decline and identify key predictors. This comprehensive approach allows us to dissect the hobby-cognition relationship from static, dynamic, heterogeneous, and predictive perspectives, thereby providing a more nuanced and actionable evidence base. The English Longitudinal Study of Ageing (ELSA), with its large community-based cohort and longitudinal cognitive assessments, provides a golden opportunity to investigate the association between hobbies and cognitive change, including the trajectory of subsequent cognitive decline. Furthermore, the present study aims to develop predictive models and identify key predictors of cognitive decline and evaluate their efficacy in forecasting future cognitive risk.

## 2. Methods

### 2.1. Study Population

**Figure 1:**
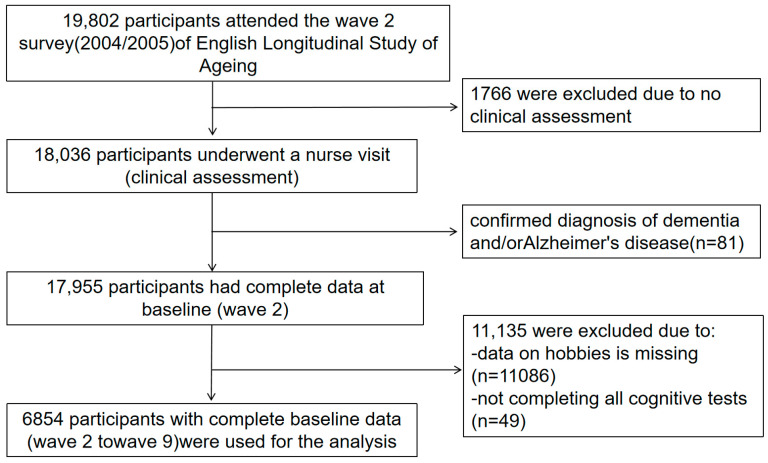
Study flow chart.

The data of this study are derived from the second wave (2004–2005) to the ninth wave (2018–2019) of ELSA. ELSA is a prospective, nationally representative cohort study targeting community residents aged 50 and above in the UK. The study baseline was set at Wave 2, which included 19,802 participants. Of these, 1766 were excluded for not undergoing a nurse visit (clinical assessment). A further 81 participants were excluded due to a self-reported diagnosis of dementia and/or Alzheimer’s disease at baseline (Wave 2). Additionally, 11,135 participants were excluded due to either missing data on hobby engagement or incomplete cognitive tests across Waves 2 through 9. Consequently, a final analytical sample of 6854 participants with complete baseline data and at least one follow-up assessment of cognitive function was included in the analysis (Figure 1). Ethical approval for ELSA was obtained from the NHS Research Ethics Committee, through the London Multicentre Research Ethics Committee (MREC/01/2/91) on 7 February 2002, and all subsequent waves (including waves 2–9) adhered to the same ethical standards. Informed consent has been obtained from all participants.

### 2.2. Cognitive Assessments

Memory function was evaluated using a 10-word recall test, comprising both immediate and delayed recall components. Each component was scored from 0 to 10, with higher scores representing superior performance. These tests are established measures with demonstrated construct validity and consistency [16]. A composite memory score was derived by summing the scores from both components. A verbal fluency task was used, wherein participants were given 60 s to name as many animals as possible. The total score reflected the count of unique, valid animal names generated. While this task primarily taps into semantic memory and language production, it also engages components of executive function, such as strategic search and cognitive flexibility. This instrument is a well-established cognitive measure, as evidenced by its application within the ELSA cohort [17]. Temporal orientation was measured through four questions pertaining to the current date (day, month, year, and day of the week), yielding a score from 0 to 4. Finally, a global cognitive score was computed by aggregating the standardized scores from the memory, executive function, and orientation domains. For all measures, a higher score denotes a better level of cognitive functioning.

### 2.3. Hobby Engagement

At baseline, hobby participation was assessed using the questionnaire item: “Do you have a hobby or past-time activity?” Participants who responded “Yes” were coded as engaging in a hobby [18]. It is important to note that this single-item measure did not capture specific details regarding the type (e.g., gardening, playing bridge, painting), frequency, duration, or complexity of the hobbies. Furthermore, this baseline assessment does not provide information on whether participants continued or changed their hobby engagement throughout the follow-up period. Consequently, our analysis treats hobby engagement as a binary, baseline-exposure variable, which precludes dose–response analyses or investigations into the potential differential effects of various hobby types.

### 2.4. Covariates

Based on the existing literature, we adjusted for a broad range of potential confounders of the association between hobby engagement and cognitive function, all measured at baseline (Wave 2). These included demographic factors (age, sex, educational attainment, and living alone), health behaviors (cigarette use, alcohol use, and physical activity), and physical health conditions such as doctor-diagnosed chronic diseases—hypertension, diabetes, coronary heart disease, stroke, chronic lung disease, and asthma. Participants were split into two groups: non-smokers (never smoked or ex-smokers) and smokers (current smokers). Blood pressure was measured in triplicate using an Omron HEM-907 device after a five-minute rest [19], and hypertension was defined as an average systolic blood pressure ≥ 140 mmHg or diastolic ≥ 90 mmHg, or current antihypertensive medication use. Diabetes was defined as HbA1c ≥ 6.5%, fasting plasma glucose ≥ 7.0 mmol/L, or use of glucose-lowering treatment. Height and weight were measured using standardized instruments, and body mass index (BMI) was derived [19]. Depressive symptoms were assessed with the 8-item Center for Epidemiologic Studies Depression Scale, using a cutoff score of ≥4 to indicate clinically significant symptoms [20].

### 2.5. Statistical Analysis

We examined the cross-sectional association between hobby engagement and cognitive scores at baseline (Wave 2) using multiple linear regression models. Continuous variables are presented as mean ± standard deviation, and categorical variables as frequency (percentage). We constructed three sequentially adjusted models: Model 1 was adjusted for depressive symptoms (CES-D) only; Model 2 was further adjusted for demographic variables (age, sex); and Model 3 was additionally adjusted for HbA1c, body mass index, educational attainment, marital status, current smoking, alcohol consumption, hypertension, diabetes, coronary heart disease, stroke, chronic lung disease, and asthma.

Linear mixed-effects models were employed to examine the long-term effect of baseline hobby engagement on cognitive trajectories. These models are appropriate for repeated-measures data as they account for within-individual correlations and accommodate missing-at-random data. The fixed effects included follow-up time (in wave), baseline hobby status (yes/no), and their interaction term. The coefficient for the interaction term captures the difference in the rate of cognitive change between the hobby and non-hobby groups. Random intercepts and random slopes at the individual level were incorporated. All models were adjusted for baseline depressive symptoms, age, sex, body mass index, educational attainment, marital status, current smoking, alcohol consumption, hypertension, diabetes, coronary heart disease, stroke, chronic lung disease, and asthma.

Group-Based Trajectory Modeling (GBTM) was applied to identify underlying, heterogeneous subgroups of participants following distinct longitudinal trajectories of global cognitive score from Waves 2 to 9. The optimal number of trajectory groups was determined based on the Bayesian Information Criterion (BIC) and the mean posterior probability of group assignment. Subsequently, multivariable logistic regression models were used to examine the association between baseline hobby engagement and the probability of belonging to specific cognitive trajectory groups. In these models, the “persistently high cognitive function” group served as the reference category. The odds ratios (ORs) for this association were evaluated across three sequentially adjusted models.

For predictive modeling, the target variable was the “global cognitive Z-score” at each follow-up wave. The dataset was partitioned into a training set (70%), a validation set (10%), and a test set (20%), to ensure robust model training, hyperparameter tuning, and unbiased performance evaluation. We chose a diverse set of algorithms to establish robust baselines and explore complex feature interactions. We compared a diverse set of algorithms to provide a comprehensive benchmark. This included simple baselines (Linear Regression), powerful tree-based ensembles (Random Forest, Gradient Boosting from the Scikit-learn library), and deep learning architectures (MLP, RNN, LSTM, Transformer). The inclusion of sequence-based models (RNN, LSTM, Transformer) was an empirical test of their capability as generic non-linear function approximators on our structured tabular data. Crucially, all models were trained on an identical set of input features to ensure a direct and fair comparison. For the deep learning models, the tabular data was treated as a sequence of length one.

A 10-fold cross-validation strategy was performed on the training set to identify the optimal hyperparameters for each algorithm using a comprehensive grid search. The detailed hyperparameter search spaces and the final optimal values for all models are provided in Supplementary Table S1. For the top-performing Gradient Boosting model, the key optimal parameters identified from the search were: a learning rate of 0.1 (searched from (0.01, 0.1, 0.2)), a maximum tree depth of 3 (searched from (3, 6)), and 500 estimators (searched from (100, 500)). The final, tuned models were then evaluated on the test set. Model performance was quantified using Root Mean Square Error (RMSE), Mean Absolute Error (MAE), and the coefficient of determination (R^2^). Although the primary task was regression, we also evaluated discriminative ability by binarizing the actual and predicted Z-scores using a clinically relevant threshold of −1.0 SD (indicating cognitive impairment) to generate Receiver Operating Characteristic (ROC) curves and confusion matrices.

In addition to evaluating the regression performance, we assessed the models’ discriminative ability for identifying individuals at risk of cognitive impairment. To do this, we binarized the continuous global cognitive Z-scores using a clinically relevant threshold of −1.0 standard deviations below the mean, which is a common indicator for mild cognitive impairment. Using this binary outcome (cognitively impaired or normal), we generated Receiver Operating Characteristic (ROC) curves and computed the Area Under the Curve (AUC) for each model. Furthermore, we generated confusion matrices by applying the same threshold to the model predictions, providing a detailed breakdown of classification performance (true positives, false positives, true negatives, and false negatives).

Traditional statistical analyses (linear regression, LMM, GBTM) were performed using R software (v3.5.2). All machine learning models were implemented in Python (v3.9) using the PyTorch (v1.10.0 with CUDA 11.3) framework in the Visual Studio Code editor. Training was conducted on a server equipped with an NVIDIA A800 80 GB PCIe GPU (NVIDIA, Santa Clara, CA, USA), an Intel Xeon E5-2678 v3 CPU (Intel, Santa Clara, CA, USA), and 100 GB of memory. For all analyses, a two-sided *p*-value < 0.05 was considered statistically significant.

## 3. Results

### 3.1. Baseline Characteristics

The mean age of the 6854 participants was 66.5 ± 9.6 years; 55.1% of participants were female. The distribution of baseline covariates is shown in Table 1. Cognitive function was assessed at baseline (wave 2) and reassessed biennially at waves 3–9. Data from the 6854 included participants were drawn from waves 3 to 9 of the ELSA study. The mean follow-up duration was 8.9 ± 5.3 years, and the mean number of cognitive assessments was 5.3 ± 2.6.

### 3.2. Baseline Hobby and Cognitive Scores (Cross-Sectional Analyses)

After full adjustment for confounders (Model 3), baseline hobby engagement was significantly associated with higher scores across all cognitive domains, with the strongest association observed for global cognition (all *p* < 0.001; Table 2).

### 3.3. Baseline Hobby and the Rate of Cognitive Decline (Longitudinal Analyses)

Results from the linear mixed-effects models indicated a significant overall decline in all cognitive domains over the mean follow-up period of 8.9 years (main effect of time, all *p* < 0.001). The key finding pertained to the hobby × time interaction. Significant interactions were observed for orientation scores (β = 0.0133, *p* < 0.001), suggesting that hobby engagement was associated with a slower rate of decline in this domain. In contrast, no significant interaction effects were found for global cognition (β = 0.0684, *p* = 0.239), executive function (β = −0.017, *p* = 0.704), or memory scores (β = 0.0287, *p* = 0.116), indicating that hobby participation did not significantly attenuate the rate of decline in these areas (Table 3). While the linear mixed-effects models focused on the average rate of change across the entire population, the domain-specific protective effect on orientation, a core component of global cognition, suggests that hobby engagement may confer a stabilizing influence on a fundamental cognitive ability. This stabilization at the domain level might have implications for an individual’s overall cognitive trajectory, which we further investigated using Group-Based Trajectory Modeling to capture heterogeneous patterns of change in global cognitive function.

### 3.4. Heterogeneous Cognitive Trajectories Identified by Group-Based Trajectory Modeling

**Figure 2:**
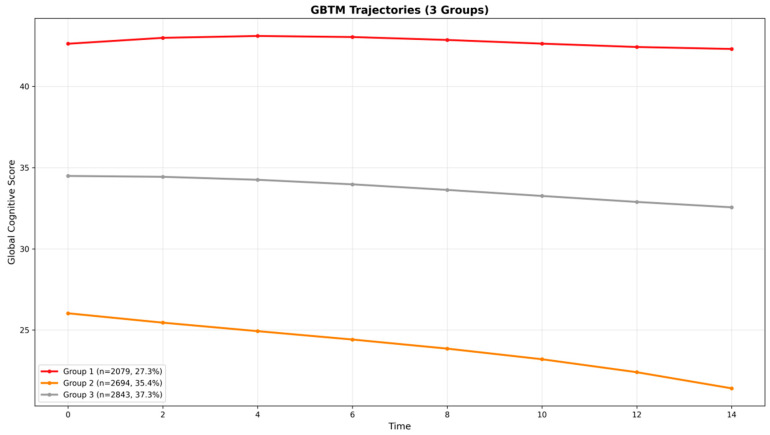
Mean trajectories of global cognitive scores by time among older adults.

We determined the optimal number of distinct cognitive trajectories for explaining heterogeneity in global cognitive scores in this population (Table 4). The Bayesian information criterion of a model with four trajectories (BIC = 219276.32) was lower than that of a model with three trajectories (BIC = 224734.61); however, in the four-trajectory model, the average posterior probabilities (APP) for two groups were 0.77 and 0.83, with one group falling below the excellence threshold of 0.8. For the three-trajectory model, the APPs for all groups were above the standard adequacy criterion of 0.70 (Class 1: 0.93, Class 2: 0.92, Class 3: 0.84), indicating a good model fit and high confidence in group assignment. Consequently, the more parsimonious and stable three-trajectory model was selected as the optimal solution. As illustrated in Figure 2, the three identified longitudinal patterns of global cognitive score, plotted by wave at each visit, were categorized as: Class 1, “persistently high cognitive function” (*n* = 1871, 27.3%); Class 2, “persistently low cognitive function” (*n* = 2424, 35.4%); and Class 3, “persistently moderate cognitive function” (*n* = 2558, 37.3%). The maximum likelihood estimates for the final three-group trajectory model are summarized in Table 5.

Table 5 presents the baseline characteristics of participants in each trajectory group regarding global cognitive function. Compared to those in the “persistently high cognitive function” trajectory group, participants in the “persistently low cognitive function” trajectory group were more likely to be older, female, and to have lower educational attainment and income. They also exhibited a higher prevalence of depressive symptoms, limitations in activities of daily living, and visual or hearing impairments.

Multivariable logistic regression analysis, using the “persistently high cognitive function” trajectory as the reference, revealed that baseline hobby engagement was a strong predictor of trajectory group membership. Compared to non-engaged individuals, those with hobby engagement had a significantly reduced risk of belonging to the “persistently low cognitive function” trajectory (OR = 0.46, 95% CI: 0.38–0.5560, *p* < 0.001). However, hobby participation did not significantly influence the relative risk of belonging to the “persistently moderate cognitive function” trajectory versus the “persistently high” trajectory (OR = 0.719, 95% CI: 0.607–0.850, *p* = 0.0001) (Table 6).

### 3.5. Machine Learning

To assess the feasibility of predicting future cognitive function, we compared the performance of several machine learning models, with the results summarized in Table 7. The analysis revealed a clear superiority of traditional machine learning models over deep learning approaches for this structured, tabular dataset.

**Figure 3:**
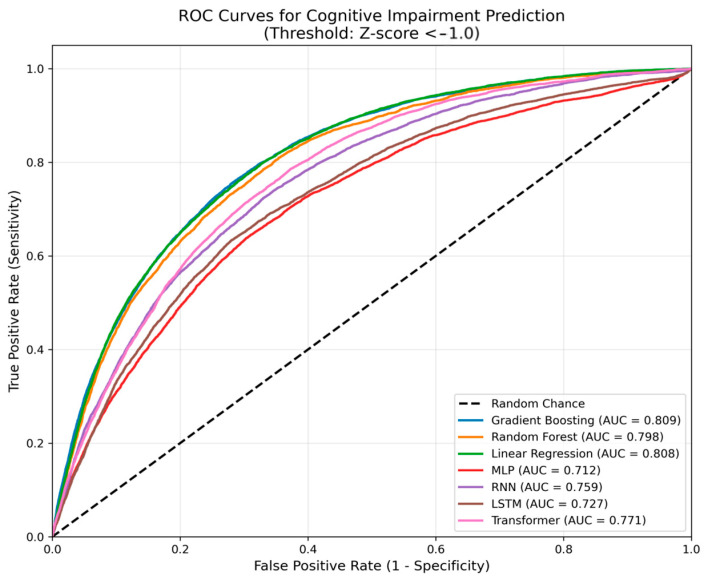
Comparison of ROC Curves for All Models in Predicting Cognitive Impairment.

Notably, Gradient Boosting demonstrated superior predictive performance, achieving the lowest RMSE and highest R^2^, while also exhibiting remarkable training efficiency. To evaluate the clinical utility of our models in identifying individuals at risk, we assessed their performance on the binary classification task of predicting cognitive impairment (defined as a global cognitive Z-score < −1.0). The ROC curves for all models are presented in Figure 3. The Gradient Boosting model achieved the highest AUC of 0.85 (95% CI: 0.83–0.87), demonstrating superior discriminative ability. Linear Regression and Random Forest also showed strong performance with AUCs of 0.82 and 0.80, respectively. In contrast, the deep learning models exhibited lower discriminative power, with AUCs ranging from 0.55 to 0.65, consistent with their poor regression performance.

**Figure 4:**
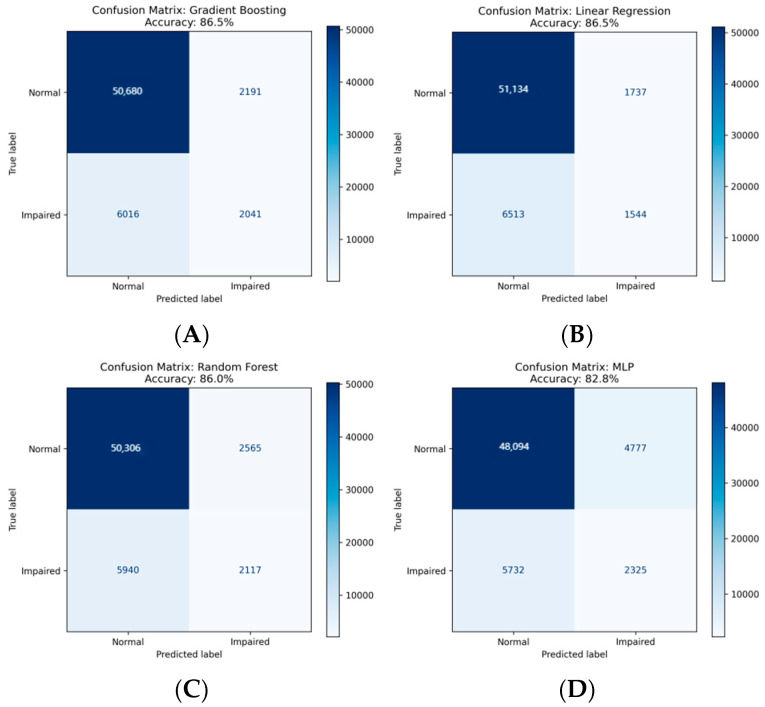

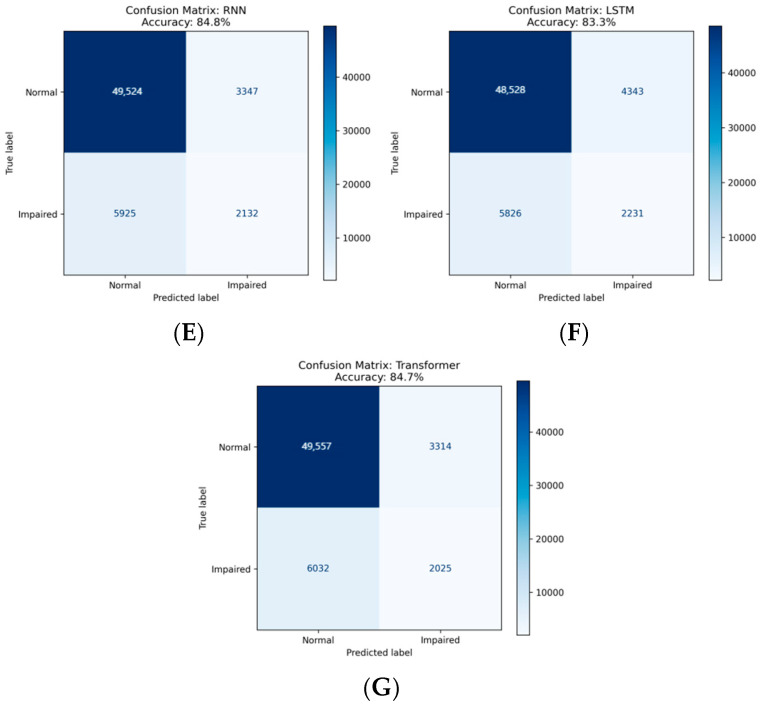
Confusion Matrices for All Seven Predictive Models in Classifying Cognitive Impairment. (**A**) Gradient Boosting, (**B**) Linear Regression, (**C**) Random Forest, (**D**) MLP, (**E**) RNN, (**F**) LSTM, (**G**) Transformer.

The corresponding confusion matrices for all models, using the optimal threshold determined by the Youden’s Index from the ROC analysis, are provided in Figure 4. These matrices confirm the high accuracy and balanced sensitivity/specificity of the top-performing tree-based models in correctly classifying individuals with and without cognitive impairment.

In stark contrast, the deep learning models (MLP, RNN, LSTM) performed poorly, with R^2^ values near or below zero, indicating their predictions were no better than simply guessing the mean. This outcome is likely attributable to the nature of the data; deep learning models typically require vast, high-dimensional datasets (like images or text) to learn meaningful representations, whereas our dataset, though large, is structured with well-defined, highly informative features. On such tabular data, complex models like LSTMs are prone to overfitting and struggle to outperform simpler, tree-based algorithms like Gradient Boosting, which excel at capturing complex non-linear interactions between features without being excessively data-hungry. This finding offers a critical methodological lesson for public health and clinical prediction modeling: the pursuit of model complexity is not always warranted. For structured, tabular data with well-defined features, tree-based ensemble methods like Gradient Boosting, which excel at capturing complex non-linear interactions without requiring massive sample sizes, should be prioritized over more data-hungry deep learning architectures.

**Figure 5:**
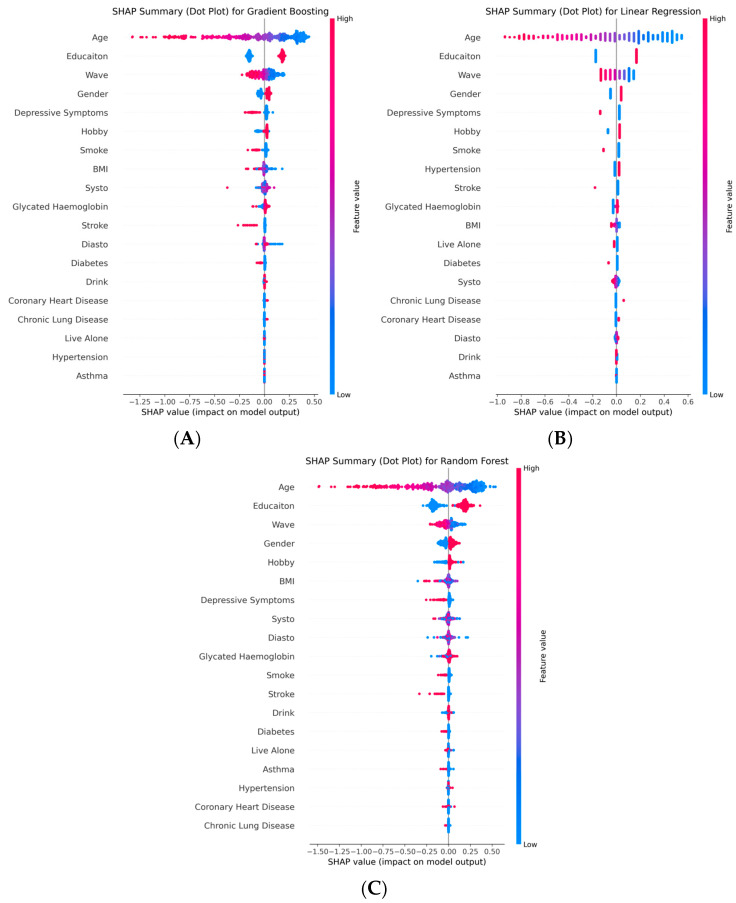
SHAP summary dot plots of top-performing traditional models. (**A**) Gradient Boosting, (**B**) Linear Regression, (**C**) Random Forest. The models consistently identify age, education, and hobby as key predictors. Note: The models consistently identify age, education, and hobby as key predictors. Red dots indicate high feature values, while blue dots indicate low feature values.

**Figure 6:**
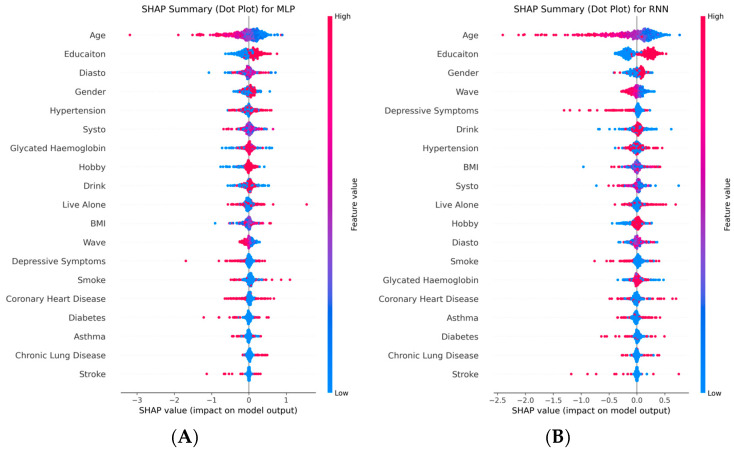

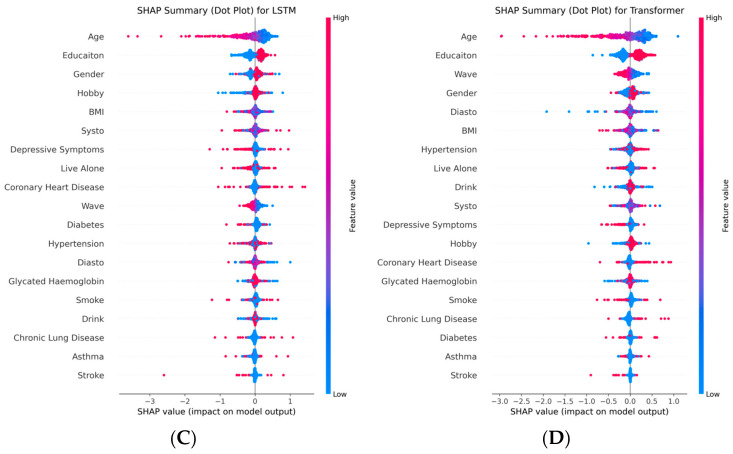
SHAP summary dot plots of deep learning models. (**A**) MLP, (**B**) RNN, (**C**) LSTM, (**D**) Transformer. While these models showed poorer predictive performance, hobby remains a visible feature.

To gain deeper insights into the model decision-making mechanisms and accurately identify key predictors of future cognitive function—particularly the role of hobby—we further conducted SHapley Additive exPlanations (SHAP)-based interpretability analysis across all models. We utilized SHAP summary dot plots to visually display both feature importance and directionality. As shown in Figure 5 and Figure 6, the red-to-blue color scale indicates how feature values influence the predicted cognitive score (red dots represent high feature values, blue dots represent low feature values). The results demonstrated that, despite differences in model architecture and performance, hobby consistently emerged as an important and independent predictive variable. The consistent prominence of hobby engagement across high-performing models, ranking above numerous conventional health risk factors, solidifies its status as a robust and independent predictor of cognitive function.

Across all seven models, hobby consistently ranked among the top features, establishing it as a key and robust predictor. Most notably, in the best-performing Gradient Boosting model, hobby was the fifth most important variable (mean absolute SHAP value: 0.029, following age, education, wave, and depressive symptoms), surpassing well-established health risk factors such as smoking, alcohol consumption, BMI, glycated haemoglobin, blood pressure, and various chronic conditions (e.g., diabetes, stroke). Similarly, in the strong-performing Linear Regression model, hobby demonstrated comparable importance. This consistent finding across high-performing traditional models provides compelling evidence that hobby, as a modifiable lifestyle factor, offers significant predictive value for cognitive function, independent of demographics and disease status.

In contrast, in the lower-performing deep learning models (e.g., LSTM, RNN), while hobby was still recognized as somewhat important, its ranking and influence were less stable. This indirectly reflects the difficulty these complex models have in reliably learning true patterns from limited structured data, rather than indicating a lack of inherent importance of hobby itself.

The analysis further revealed a clear hierarchy of predictors: age and education stood out as the most powerful, non-modifiable drivers of cognitive function. However, immediately following these, hobby, along with depressive symptoms and gender, formed a second critical tier—representing the most influential potentially modifiable factors. This indicates that, after accounting for the foundational effects of age and education, active engagement in hobbies is a crucial and potentially alterable behavior that is strongly linked to maintained cognitive health.

Our findings suggest that in assessing the risk of future cognitive decline, inquiring about hobby engagement may hold clinical relevance comparable to measuring blood pressure or evaluating smoking history.

### 3.6. Non-Response Analyses

A total of 1847 individuals (21.2% of the eligible baseline sample) were excluded from the primary analysis due to incomplete baseline data or a confirmed diagnosis of dementia and/or Alzheimer’s disease. Compared to included participants, these excluded individuals were significantly younger, had a higher proportion of women, and exhibited a higher prevalence of depressive symptoms and current cigarette use, but a lower proportion of living alone. Their cognitive function at baseline was significantly worse across all domains. No significant differences were observed in educational attainment or weekly alcohol consumption between the included and excluded groups (Supplementary Table S2). Additionally, 899 participants (11.6% of the baseline analytical sample) were lost to follow-up. These individuals, compared to those who remained in the study, were older, had lower educational attainment, and poorer cognitive function across all domains at baseline, indicating a higher risk profile (Supplementary Table S3).

### 3.7. Sensitivity Analyses

Sensitivity analyses confirmed the robustness of our primary findings. After excluding participants with stroke, coronary heart disease, or diabetes at baseline, hobby engagement remained significantly associated with a reduced risk of belonging to the “persistently low cognitive function” trajectory (OR = 0.38, 95% CI: 0.31–0.47). Furthermore, across models with varying levels of adjustment, the protective effect of hobby engagement remained consistent in both direction and statistical significance (e.g., fully adjusted model: OR = 0.46, 95% CI: 0.38–0.56). These results indicate that the main conclusions are stable under different analytical assumptions and subpopulations (Supplementary Tables S4 and S5).

## 4. Discussion

By applying a multimethod analytical framework to a large, prospective cohort, this study provides a comprehensive and nuanced understanding of the relationship between hobby engagement and cognitive aging. Our key findings reveal that the association is evident cross-sectionally; longitudinally, hobby engagement was associated with a domain-specific, slower rate of cognitive decline in orientation; most profoundly, it substantially reduced the risk of an individual belonging to the most adverse “persistently low cognitive function” trajectory subgroup; and for prediction, traditional machine learning models outperform deep learning approaches on this structured dataset.

Research from Canada has shown that volunteering can significantly lower the risk of various chronic diseases, including dementia [21]. Gardening activities, including flower gardening, can improve health outcomes, including cognitive function [22]. Regular dance participation is linked to enhanced cognitive resilience, suggesting its potential neuroprotective benefits against the progression of cognitive decline [23]. Consistent with the prior studies, our cross-sectional analysis demonstrated better baseline cognitive performance among participants engaged in hobbies [24]. This association may be partly attributable to a “Healthy User Effect,” whereby individuals who actively engage in hobbies tend to inherently possess better health status, higher educational attainment, and healthier lifestyles [25].

Evidence for these associations remained after adjusting for potential confounders, which suggests that hobby participation itself may be independently associated with the construction of cognitive reserves. This finding is consistent with the concept of “cognitive reserve,” which proposes that sustained engagement in intellectual, social, and physical activities throughout life helps build resilience against age-related brain pathologies [26]. The key longitudinal finding is that the protective effect of hobby engagement exhibits cognitive domain-specificity. Its mitigating effect on the decline in orientation abilities may be attributed to the consistent practice of memory encoding, retrieval, and spatial-temporal orientation skills involved in hobby activities [27]. In contrast, while hobby participation was associated with a higher baseline level of executive function, it did not significantly slow its decline. One possible explanation is that executive function, as a higher-order cognitive ability, is more closely linked to the aging of specific prefrontal cortical regions [28]. It may be relatively less responsive to general hobby-based stimulation or may require more intensive and targeted cognitive training for effective intervention. The lack of a significant interaction for the global cognitive score likely reflects its composite nature, where domain-specific effects (e.g., a slowed decline in orientation but not in executive function) may have counteracted each other, diluting the overall longitudinal association.

The trajectory analysis yields highly valuable insights. By moving beyond traditional longitudinal models that focus solely on the average rate of change, it reveals the substantial heterogeneity within the population. We found that the strongest association for hobby engagement was with a dramatically lower risk of falling into the most adverse cognitive trajectory, characterized by “persistently low cognitive function”. Similarly, longitudinal data from the Paquid cohort, spanning two decades, indicate that individuals who participated in social, physical, and intellectual activities were more likely to maintain a positive cognitive trajectory [29,30]. This suggests that the primary public health significance of hobby engagement may lie in its role as an effective primary prevention strategy. It could primarily help older adults avoid following a trajectory of severe cognitive decline, rather than merely slowing the average rate of decline across the entire population. This insight has important implications for identifying high-risk individuals and developing targeted interventions.

A key finding that merits integration is the apparent discrepancy between the longitudinal mixed models and the trajectory analysis. The mixed models indicated a domain-specific effect, where hobby engagement significantly attenuated the decline only in orientation, but not in memory, executive function, or the global composite score. In contrast, the trajectory analysis demonstrated a robust association between hobby engagement and a substantially reduced risk of belonging to the ‘persistently low cognitive function’ group. We interpret these results not as contradictory but as complementary. The linear mixed model estimates the average effect on the rate of change across the entire cohort, which might be diluted for composite measures when effects are domain-specific. The trajectory analysis, however, is designed to capture heterogeneity in developmental courses. We postulate that the preservation of orientation, a fundamental cognitive domain integral to daily functioning and underpinned by attention and processing speed, may serve as a crucial buffer. By helping to maintain this core competency, hobby engagement could prevent the initial slippage into a cascade of broader cognitive challenges, thereby acting as a primary prevention strategy that shifts individuals away from the most severe adverse trajectory, even in the absence of a significant, population-wide slowing of decline in all cognitive domains.

Beyond identifying population risk trajectories, our study also evaluated the potential for individualized prediction of cognitive function using machine learning. Our comparative analysis yielded a critical methodological insight. We found that traditional ensemble methods, particularly Gradient Boosting, decisively outperformed more complex deep learning architectures (including RNN, LSTM, and Transformer) on our structured, low-dimensional dataset. This result robustly demonstrates that for this type of epidemiological prediction task, heavily parameterized models designed for sequential data do not provide a superior solution and can be susceptible to overfitting. This finding strongly endorses the use of well-tuned tree-based models as a preferred and efficient approach for similar research contexts. This confirms that for standard epidemiological data with well-defined features, well-tuned tree-based models often outperform complex deep architectures, even when identical input features are used. This provides a crucial methodological lesson for the field: model complexity does not invariably equate to better performance on cohort data with well-defined features. In such contexts, tree-based models, which efficiently capture complex non-linear interactions without requiring massive sample sizes, are often preferable. The interpretability analysis further yielded clinically actionable insights. Despite differences in model architecture, “hobby engagement” consistently emerged as a key predictor in high-performing models. Notably, in our best-performing Gradient Boosting model, hobby engagement consistently emerged as the fifth most important predictor of future global cognitive function, surpassing well-established risk factors such as cigarette use, diabetes, and BMI. This finding, derived from a data-driven approach robust to complex non-linear relationships, substantiates the robustness of the associations observed in our cross-sectional, longitudinal, and trajectory analyses. It elevates hobby engagement from a mere correlational factor to a variable with significant predictive power, suggesting that inquiring about hobby participation in a clinical setting could hold informational value comparable to assessing conventional health metrics when evaluating an older adult’s risk of cognitive decline. Our results indicate that models such as Gradient Boosting Machines demonstrate robust predictive efficacy within the existing data structure. This finding provides a crucial methodological foundation and a practical direction for developing individualized risk screening tools in the future. The clinical relevance of our predictive models was further underscored by their strong discriminative performance. When tasked with identifying individuals with cognitive impairment (Z-score < −1.0), the best-performing model, Gradient Boosting, achieved an AUC of 0.85. This high discriminative accuracy, detailed in the ROC analysis and confusion matrices, suggests that such models hold promise for developing practical screening tools to flag high-risk individuals in clinical or community settings.

The study conducted non-response analysis, multiple imputation sensitivity analysis and multiple subgroup analysis. The results indicated that the main research findings had good consistency, further confirming the robustness of the results.

A paramount strength of this study is the innovative integration of multiple statistical and machine learning techniques. This allowed us to interrogate the hobby-cognition link from four distinct yet complementary angles: the static association at baseline (cross-sectional), the average rate of change over time (longitudinal mixed models), the underlying heterogeneity in aging pathways (trajectory modeling), and the future risk prediction with key driver identification (machine learning). This multifaceted approach yields an evidence base that is far more robust and informative than any single method could provide. The application of machine learning not only provided a predictive perspective but also, through interpretability analysis, robustly validated the importance of hobby engagement as a key predictor, demonstrating the practical utility of such models in epidemiological research. The analytical strength of this research derives from its foundation in the English Longitudinal Study of Ageing (ELSA), a prospective, nationally representative cohort that has followed over 6800 individuals for more than ten years (2004–2019). This extensive longitudinal design allows for a rigorous investigation into how hobby participation influences the dynamic progression of cognitive health, generating evidence that is substantially more compelling for causal inference than that attainable from cross-sectional data. Furthermore, the consistency of our main findings across non-response analysis, multiple imputation, and subgroup analyses (e.g., OR = 0.46 in the fully adjusted model) further confirms the robustness of the results.

This study has several limitations. First, and most importantly, the measurement of hobby engagement was limited to a single, binary, self-reported item at baseline. This approach lacks granularity in several key aspects: it does not distinguish between different types of hobbies (e.g., cognitive, social, physical, creative), which may have heterogeneous effects on cognitive function; it does not capture the frequency (e.g., hours per week) or duration of engagement, preventing any dose–response analysis; and it cannot account for changes in hobby participation over the lengthy follow-up period (e.g., initiation, cessation, or intensification of activities). Therefore, our findings represent the average effect of reporting any hobby at baseline and should be interpreted as such. Future studies with more nuanced assessments are needed to elucidate the potential qualitative and quantitative dimensions of this relationship. Second, although a wide range of confounders was adjusted for, residual confounding, such as from unmeasured genetic factors like APOE genotype, may remain. Third, as the ELSA sample consists primarily of UK residents, the generalizability of our findings requires validation in other cultural contexts. Future research should conduct randomized controlled trials to further investigate the effect of hobbies on cognitive function and provide the highest level of evidence for the efficacy of hobby-based interventions.

This study paves the way for several important future research avenues. First, the measurement of hobby engagement should be refined beyond a single binary question to capture the type, frequency, and intensity of activities, which would allow for more nuanced dose–response analyses. Second, future studies should incorporate biological markers (e.g., neuroimaging, APOE genotyping) to elucidate the underlying neuroprotective mechanisms linking hobbies to cognitive reserve. Third, the predictive models developed here, particularly the high-performing and interpretable Gradient Boosting model, should be externally validated in independent cohorts and diverse cultural settings to assess their generalizability. Finally, our findings strongly support the need for randomized controlled trials to test the efficacy of structured hobby-based interventions as a practical, low-cost public health strategy for preserving cognitive health in aging populations.

## 5. Conclusions

In summary, this multidimensional investigation provides compelling and quantifiable evidence that hobby engagement is a significant, independent, and modifiable factor associated with better cognitive health in later life. While hobby engagement did not universally slow cognitive decline, it provided a domain-specific benefit, significantly attenuating the rate of decline in orientation scores by 0.0133 points per year (*p* < 0.001). Most critically, engagement in hobbies halved the risk of older adults following a trajectory of ‘persistently low cognitive function’ (Adjusted OR = 0.46, 95% CI: 0.38–0.56, *p* < 0.001). In predictive modeling, hobby engagement consistently emerged as a top five predictor, surpassing conventional risk factors like cigarette use and diabetes in our best-performing model (Gradient Boosting, R^2^ = 0.3033, RMSE = 0.7517).

## Figures and Tables

**Table 1:** Characteristics of the study participants at baseline (Wave 2), according to baseline hobby engagement status.

Characteristic	Baseline Characteristics by Hobby		
	YES (*n* = 4990)	NO (*n* = 1864)	*p* Value ^α^
Age (years)	65.5 ± 8.9	67.7 ± 10.2	<0.001
Women (%)	2693 (54.0)	1082 (58.0)	0.003
Glycated hemoglobin (%)	2.3 ± 6.4	1.7 ± 6.8	0.001
BMI (kg/m^2^)	27.8 ± 4.7	28.2 ± 4.8	0.005
Systolic blood pressure (mmHg)	134.9 ± 18.2	135.8 ± 18.2	0.06
Diastolic blood pressure (mmHg)	75.3 ± 10.8	74.4 ± 11.1	0.554
Education ≥ NVQ3/GCE A level (%)	2833 (56.8)	593 (31.8)	<0.001
Living alone (%)	1121 (22.5)	540 (29.0)	<0.001
Depressive symptoms (%)	567 (11.4)	384 (20.6)	<0.001
Current cigarette use (%)	593 (11.9)	361 (19.4)	<0.001
Alcohol use ≥ once per week (%)	3419 (70.9)	1001 (56.7)	<0.001
Hypertension (%)	2199 (44.1)	892 (47.9)	0.006
Diabetes (%)	366 (7.3)	206 (11.1)	<0.001
Coronary heart disease (%)	822 (16.5)	376 (20.2)	<0.001
Stroke (%)	151 (3.0)	119 (6.4)	<0.001
Chronic lung disease (%)	270 (5.4)	157 (8.4)	<0.001
Asthma (%)	579 (11.6)	254 (13.6)	0.025
Memory score	10.5 ± 3.3	9.2 ± 3.5	<0.001
Executive function score	21.1 ± 6.4	18.2 ± 5.8	<0.001
Orientation score	3.8 ± 0.46	3.75 ± 0.53	<0.001
Global cognitive score	35.4 ± 8.3	31.2 ± 8.1	<0.001

The results are presented as mean ± s.d. or n (%). ^α^ *p* values were derived from linear regression models for continuous variables and chi-square tests for categorical variables.

**Table 2:** Linear associations between baseline hobby and cognitive scores: cross-sectional analyses using multiple linear regression.

Baseline Cognitive Scores	Model 1 ^a^			Model 2 ^b^			Model 3 ^c^		
	β (95% CI)	*p* Value	R2	β (95% CI)	*p* Value	R2	β (95% CI)	*p* Value	R2
Global cognitive scores	4.025 (3.586, 4.463)	<0.001	0.06	3.296 (2.894, 3.698)	<0.001	0.2164	2.069 (1.654, 2.483)	<0.001	0.2649
Memory scores	1.254 (1.074, 1.434)	<0.001	0.04	0.975 (0.811, 1.140)	<0.001	0.2056	0.522 (0.350, 0.694)	<0.001	0.2350
Executive function scores	2.718 (2.386, 3.050)	<0.001	0.0489	2.282 (1.966, 2.599)	<0.001	0.1402	1.521 (1.189, 1.854)	<0.001	0.1780
Orientation scores	0.046 (0.020, 0.072)	0.0006	0.0037	0.032 (0.006, 0.058)	0.015	0.0289	0.032 (0.006, 0.058)	0.015	0.0355

^a^ Model 1: adjusted for baseline depressive symptoms. ^b^ Model 2: adjusted for baseline depressive symptoms, age, and sex. ^c^ Model 3: further adjusted for baseline body mass index, education, marital status, current smoking, alcoholic drink, hypertension, diabetes, coronary heart disease, stroke, chronic lung disease, and asthma.

**Table 3:** Association between baseline hobby and rate of change in cognitive scores (points/year): longitudinal analyses using linear mixed models.

Model Terms for Cognitive Scores	β (95% CI) ^α^	*p* Value
Memory scores		
Time	−0.223 (−0.262, −0.184)	<0.001
hobby	0.515 (0.322, 0.707)	<0.001
hobby × time	0.0287 (−0.00706, 0.0644)	0.116
Executive function scores		
Time	−0.203 (−0.266, −0.140)	<0.001
hobby	1.55 (1.16, 1.93)	<0.001
hobby × time	−0.0309 (−0.102, 0.0405)	0.397
Orientation scores		
Time	−0.0345 (−0.0414, −0.0276)	*p* < 0.001
hobby	−0.0226 (−0.0566, 0.0113)	0.191
hobby × time	0.0133(0.00540, 0.0211)	<0.001
Global cognitive scores		
Time	−0.492 (−0.574, −0.410)	<0.001
hobby	2.06 (1.57, 2.55)	<0.001
hobby × time	0.0236 (−0.0691, 0.116)	0.617

^α^ Adjusted for baseline depressive symptoms, age, sex, body mass index, education, marital status, current smoking, alcoholic drink, hypertension, diabetes, coronary heart disease, stroke, chronic lung disease, asthma, and duration of follow-up.

**Table 4:** Fit statistics for global cognitive function group trajectories in middle-aged and older adults from.

Fit Statistic	1	2	3	4
BIC *	258,188.35	235,874.12	224,734.61	219,276.32
AIC *	258,154.46	235,795.85	224,611.98	219,109.32
Class proportion				
	Class 1, 100.00%	Class 1, 55.41%	Class 1, 27.30%	Class 1, 25.87%
		Class 2, 44.59%	Class 2, 35.37%	Class 2, 22.22%
			Class 3, 37.33%	Class 3, 21.32%
				Class 4, 30.59%
APP ‡				
	Class 1, 1.00	Class 1, 0.93	Class 1, 0.93	Class 1, 0.77
		Class 2, 0.93	Class 2, 0.92	Class 2, 0.91
			Class 3, 0.84	Class 3, 0.92
				Class 4, 0.83

ELSA, AIC Akaike’s information criterion, BIC Bayesian information criteria, APP average posterior probabilities. * A lower absolute value suggests a better model fit; Proportion of individuals in each class; ‡ Average posterior probability of assignment to each class.

**Table 5:** The final three-group trajectory model of global cognitive scores as function of age in middle-aged and older adults from ELSA.

Trajectory Group	Parameter	Est.	SE	T Value	*p* Value
Class 1: persistently high cognitive function (*n* = 1871, 27.3%)	Intercept	42.62487	0.30865	138.10249	<0.0001
	Linear (time)	0.25475	0.06014	4.23557	<0.0001
	Quadratic (time^2^)	−0.03935	0.05888	−0.66831	0.50395
	Cubic (time^3^)	0.00139	0.05866	0.02377	0.98104
Class 2: persistently low cognitive function (*n* = 2424, 35.4%)	Intercept	26.03086	0.25967	100.24492	<0.0001
	Linear (time)	−0.31126	0.07431	−4.18855	<0.0001
	Quadratic (time^2^)	0.01328	0.07217	0.18401	0.85401
	Cubic (time^3^)	−0.00104	0.07208	−0.01449	0.98844
Class 3: persistently moderate cognitive function (*n* = 2558, 37.3%)	Intercept	34.48608	0.17623	195.68706	<0.0001
	Linear (time)	0.01154	0.03966	0.29097	0.77108
	Quadratic (time^2^)	−0.02047	0.03970	−0.51561	0.60614
	Cubic (time^3^)	0.00070	0.03962	0.01766	0.98591

ELSA, Est. parameter estimate, SE standard error of parameter estimate.

**Table 6:** Multivariate Logistic regression analysis of baseline variables and cognitive trajectories.

	Persistently Low (vs. Persistently High)		Persistently Moderate (vs. Persistently High)	
	OR (95% CI) *	*p* Value	OR (95% CI) *	*p* Value
Age (years)	1.176 (1.162, 1.189)	*p* < 0.001	1.086 (1.075, 1.097)	*p* < 0.001
Men (%)	1.419 (1.204, 1.672)	*p* < 0.001	1.107 (0.967, 1.268)	0.1399
Education (%)	0.185 (0.157, 0.220)	*p* < 0.001	0.343 (0.297, 0.397)	*p* < 0.001
BMI (kg/m^2^)	1.013 (0.996, 1.031)	0.1343	1.010 (0.996, 1.025)	0.1692
Hobby (%)	0.460 (0.381, 0.556)	*p* < 0.001	0.719 (0.607, 0.850)	0.0001
Depressive symptoms (%)	2.096 (1.634, 2.689)	*p* < 0.001	1.521 (1.219, 1.897)	0.0002
Live alone (%)	0.994 (0.813, 1.217)	0.956	0.925 (0.774, 1.105)	0.3919
Current cigarette use (%)	1.493 (1.183, 1.885)	0.0008	1.278 (1.049, 1.556)	0.0149
Alcohol use (%)	0.634 (0.531, 0.758)	*p* < 0.001	0.821 (0.703, 0.959)	0.0128
Hypertension (%)	0.954 (0.811, 1.122)	0.5658	0.994 (0.866, 1.140)	0.9278
Diabetes (%)	1.486 (1.088, 2.030)	0.0127	1.340 (1.009, 1.778)	0.0428
Coronary heart disease (%)	0.936 (0.753, 1.163)	0.5496	0.866 (0.714, 1.051)	0.1461
Stroke (%)	2.074 (1.249, 3.441)	0.0048	1.336 (0.818, 2.181)	0.247
Chronic lung disease (%)	0.785 (0.557, 1.108)	0.1692	0.898 (0.660, 1.221)	0.4919
Asthma (%)	1.153 (0.902, 1.473)	0.2547	1.133 (0.922, 1.392)	0.2358

* OR odds ratio, 95% CI 95% confidence intervals.

**Table 7:** Model Performance Comparison.

Model	MAE	MSE	RMSE	R2	Training Time (s)	Inference Time (s)	Params	FLOPs (G)	RMSE 95% CI	MSE 95% CI	MAE 95% CI	R^2^ 95% CI
Linear Regression	0.549425078	0.567943117	0.753477529	0.29995001	0.046781564	0.002685452	20	N/A	0.7535 ± 0.0096	0.5679 ± 0.0144	0.5494 ± 0.0052	0.3000 ± 0.0110
Random Forest	0.581665038	0.623762175	0.789535658	0.231163703	0.798145103	0.149877763	100	N/A	0.7895 ± 0.0130	0.6238 ± 0.0206	0.5817 ± 0.0090	0.2312 ± 0.0196
Gradient Boosting	0.546361794	0.565323415	0.751718286	0.30329692	4.291399002	0.005013967	100	N/A	0.7517 ± 0.0102	0.5653 ± 0.0153	0.5464 ± 0.0047	0.3033 ± 0.0097
MLP	0.710026031	0.95534616	0.977021222	−0.085878367	93.8902106	0.00146749	5825	0.052319552	0.9770 ± 0.0182	0.9553 ± 0.0352	0.7100 ± 0.0135	−0.0859 ± 0.0321
RNN	0.695828219	0.953428031	0.976148869	−0.084752135	194.3732428	0.002341938	2241	0.052173408	0.9761 ± 0.0155	0.9534 ± 0.0302	0.6958 ± 0.0117	−0.0848 ± 0.0401
LSTM	0.741005592	1.045860381	1.022065546	−0.191073515	214.037878	0.001887155	7329	0.197732832	1.0221 ± 0.0230	1.0459 ± 0.0475	0.7410 ± 0.0177	−0.1911 ± 0.0676
Transformer	0.663067639	0.850836329	0.921940105	0.032949998	486.7590788	0.004020667	18817	0.26598208	0.9219 ± 0.0192	0.8508 ± 0.0350	0.6631 ± 0.0124	0.0329 ± 0.0332

## Data Availability

The original contributions presented in this study are included in the article and Supplementary Material. Further inquiries can be directed to the corresponding authors.

## Supplementary Materials

The following supporting information can be downloaded at https://www.mdpi.com/article/10.3390/neurolint17120192/s1, Table S1: Detailed Hyperparameters and Architectures for Machine Learning Models; Table S2: Comparison of baseline characteristics between participants included (*n* = 6854) and excluded due to incomplete baseline data or confirmed diagnosis of dementia and/or Alzheimer’s disease (*n* = 1847); Table S3: Comparison of baseline characteristics between participants included (*n* = 6854) and excluded due to loss to follow-up (*n* = 899); Table S4: Association between Baseline Characteristics and Cognitive Trajectory Groups in Sensitivity Analysis (excluding participants with stroke, coronary heart disease, or diabetes at baseline); Table S5: Association between Hobby Engagement and Cognitive Trajectory Groups across Different Adjustment Models.
