# Supplementary material for: The Influence of Hobby Engagement on Cognitive Function Among Older Adults: A Population-Based Cohort Study Using Statistical Analysis and Machine Learning Predictions
Source: Neurol Int. 2025 Nov 27;17(12):192. doi: 10.3390/neurolint17120192 (PMC12736032; doi:10.3390/neurolint17120192)
Supplement: Supplementary file 1 [file neurolint-17-00192-s001.zip › neurolint-3958103-supplementary.pdf]

Table S1. Detailed Hyperparameters and Architectures for Machine Learning Models

**Model Family**	**Algorithm**	**Key Hyperparameters / Architecture Details**	**Training Settings**
Traditional ML	Linear Regression	Default settings (scikit-learn implementation)	Solver: Auto-selected based on data type
	Random Forest	Number of estimators (trees): 100<br>Max depth: 10<br>Criterion: Squared Error	Random state: 42<br>n_jobs: -1 (using all processors)
	Gradient Boosting	Number of estimators (boosting stages): 100<br>Learning rate: 0.1 (default)<br>Max depth: 3 (default)<br>Loss function: Squared Error	Random state: 42
Deep Learning	MLP (Multilayer Perceptron)	Input Layer: N features<br>Hidden Layer 1: Linear(64 units) + ReLU<br>Hidden Layer 2: Linear(32 units) + ReLU<br>Output Layer: Linear(1 unit)	Optimizer: Adam<br>Learning rate: 0.001<br>Loss function: MSELoss<br>Batch size: 32<br>Epochs: 50
	RNN (Recurrent Neural Network)	RNN Layer: hidden_size=32, num_layers=1, batch_first=True<br>Fully Connected Layer 1: Linear(32 →16) + ReLU	Optimizer: Adam<br>Learning rate: 0.001

Model Family	Algorithm	Key Hyperparameters / Architecture Details	Training Settings
		Output Layer: Linear(16→1)	Loss function: MSELoss
			Batch size: 32
			Epochs: 50
			Optimizer: Adam
		LSTM Layer: hidden_size=32, num_layers=1, batch_first=True	Learning rate: 0.001
	LSTM (Long Short-Term Memory)	Fully Connected Layer 1: Linear(32 →16) + ReLU	Loss function: MSELoss
		Output Layer: Linear(16→1)	Batch size: 32
			Epochs: 50
		Embedding Layer: Linear(N→32)	
		Encoder: 2 layers, 4 attention heads	Optimizer: Adam
		Model dimension: 32	Learning rate: 0.001
	Transformer	Feedforward dimension: 64	Loss function: MSELoss
		Pooling: AdaptiveAvgPool1d(1)	Batch size: 32
		Fully Connected: Linear(32→32) + ReLU	Epochs: 50
		Output: Linear(32→1)	

Table S2. Comparison of baseline characteristics between participants included (n=6854,and excluded due to incomplete baseline data or confirmed diagnosis of dementia and/or Alzheimer's disease(n=1847)

Characteristic	Included (n=6854)	Excluded (n=1847)	P value[α]
Age (years)	66.5 ± 9.6	62.3 ± 13	< 0.001
Women (%)	4203 (55.1)	1103 (61)	< 0.001
Education ≥ NVQ3/GCE A level (%)	3699 (53.3)	867 (51.5)	0.188
Living alone (%)	1981 (26)	352 (19.5)	< 0.001
Depressive symptoms (%)	1137 (15)	302 (18.6)	< 0.001
Current smoking (%)	1101 (14.5)	370 (20.5)	< 0.001
Alcoholic drink ≥ once per week (%)	4494 (67)	833 (64.5)	0.084
Memory scores	10 ± 3.5	9.8 ± 4	0.036
Executive function scores	19.9 ± 6.5	19.2 ± 7.4	< 0.001
Orientation scores	3.8 ± 0.5	3.7 ± 0.7	< 0.001
Global cognitive scores	33.7 ± 8.8	32.9 ± 10.1	0.002

The results are presented as mean + SD or n (%). [α] The differences between participants included and excluded were tested using the t-test or chi-square test.

Table S3.Comparison of baseline characteristics between participants included (n=6854 and excluded due to loss to follow-up (n=899)

Characteristic	Included (n=6854)	Excluded (n=899)	P value[α]
Age (years)	66.5 ± 9.6	70.1 ± 10.7	<0.001
Women (%)	4203 (55.1)	471 (52.4)	0.084
Education ≥ NVQ3/GCE A level (%)	3699 (53.3)	301 (36.7)	<0.001
Living alone (%)	1981 (26)	255 (28.4)	0.091
Depressive symptoms (%)	1137 (15)	170 (19.1)	<0.001
Current smoking (%)	1101 (14.5)	144 (16)	0.17
Alcoholic drink ≥ once per week (%)	4494 (67)	416 (59.2)	<0.001
Glycated hemoglobin (%)	2.2 ± 6.5	0.4 ± 7.5	<0.001
BMI (kg/m²)	28 ± 4.9	27.7 ± 5.1	0.199
Systolic blood pressure (mmHg)	135.1 ± 18.7	137.1 ± 20.9	0.008
Diastolic blood pressure (mmHg)	75.2 ± 11.1	73.9 ± 12.4	0.003
Diabetes (%)	556 (8.3)	82 (9.1)	0.424
Memory scores	10 ± 3.5	8.6 ± 3.8	<0.001
Executive function scores	19.9 ± 6.5	17.6 ± 6.7	<0.001

Orientation scores	3.8 ± 0.5	3.6 ± 0.7	<0.001
Global cognitive scores	33.7 ± 8.8	29.9 ± 9.5	<0.001

The results are presented as mean ± SD, median (quartile 1-quartile 3), or n (%). [α] The differences between participants included and excluded were tested using the t-test, Wilcoxon rank test or chi-square test.

Table S4. Association between Baseline Characteristics and Cognitive Trajectory Groups in Sensitivity Analysis (Excluding participants with stroke, coronary heart disease, or diabetes at baseline)

**Variable**	**Odds Ratio (95% CI)**	
	**Group 2 vs Group 1**	**Group 3 vs Group 1**
Hobby engagement	0.38 (0.31-0.47)***	0.62 (0.52-0.75)***
Age (per year increase)	1.18 (1.17-1.20)***	1.09 (1.08-1.10)***
Gender (Male vs Female)	1.34 (1.12-1.59)***	1.11 (0.96-1.28)
Education (≥NVQ3/GCE A level vs <)	0.17 (0.14-0.20)***	0.32 (0.27-0.37)***
Depressive symptoms (Yes vs No)	1.99 (1.51-2.62)***	1.44 (1.14-1.84)**

Group 1: Persistently high cognitive function (reference group); Group 2: Persistently low cognitive function; Group 3: Persistently moderate cognitive function. Model adjusted for age, gender, education, and depressive symptoms. *** $p < 0.001$, ** $p < 0.01$, * $p < 0.05$.

Table S5. Association between Hobby Engagement and Cognitive Trajectory Groups across Different Adjustment Models

Adjustment Model	Group 2 vs Group 1	Group 3 vs Group 1
Model 1 (Age + Gender)	0.28 (0.24-0.33)***	0.53 (0.46-0.62)***
Model 2 (+ Education + Depression)	0.41 (0.35-0.49)***	0.68 (0.58-0.79)***
Model 3 (Full adjustment)	0.46 (0.38-0.56)***	0.72 (0.61-0.85)***

Data are presented as odds ratio (95% confidence interval). Group 1: Persistently high cognitive function (reference group); Group 2: Persistently low cognitive function; Group 3: Persistently moderate cognitive function. *** $p < 0.001$.
